# The Glomerular Filtration Rate Estimators in the Pharmacokinetic Modelling in Acute Kidney Injury: An Observational Study

**DOI:** 10.3390/antibiotics10020158

**Published:** 2021-02-04

**Authors:** Silvijus Abramavicius, Vaidotas Galaune, Agile Tunaityte, Astra Vitkauskiene, Gintautas Gumbrevicius, Aurelija Radzeviciene, Romaldas Maciulaitis

**Affiliations:** 1 Laboratory of Preclinical Drug, Investigation Institute of Cardiology, Lithuanian University of Health Sciences, LT-47181 Kaunas, Lithuania; silvijus.abramavicius@lsmuni.lt (S.A.); vaidotas.galaune@lsmu.lt (V.G.); gintautas.gumbrevicius@lsmuni.lt (G.G.); aurelija.radzeviciene@lsmuni.lt (A.R.); romaldas.maciulaitis@lsmuni.lt (R.M.); 2 Department of Laboratory Medicine, Lithuanian University of Health Sciences, LT-47181 Kaunas, Lithuania; astra.vitkauskiene@lsmuni.lt; 3 Institute of Physiology and Pharmacology, Medical Academy, Lithuanian University of Health Sciences, LT-44307 Kaunas, Lithuania; 4 Nephrology Department, Lithuanian University of Health Sciences, LT-47181 Kaunas, Lithuania

**Keywords:** glomerular filtration rate, estimation, acute kidney injury, creatinine clearance

## Abstract

The glomerular filtration rate (GFR), according to which the drug dose for patients with chronic kidney disease (CKD) is adjusted, is computed with estimators (eGFR) that are developed specifically for CKD. These particular types of estimators are also used in population pharmacokinetic (pop PK) modelling in drug development. Similar approaches without scientific validation have been proposed for patients with acute kidney injury (AKI), yet it is uncertain which specific eGFR should be used for drug dosing or in pop PK models in patients with AKI. In our study, we included 34 patients with AKI and vancomycin (VCM) treatment, and we built both individual PK and pop PK (non-linear mixed-effects, one-compartment) models to see which eGFR estimator is the best covariate. In these models different eGFRs (Cockcroft-Gault, MDRD, CKD-EPI 2009, Jelliffe and Jelliffe, Chen et al., and Yashiro et al. 2013) were used. We included six additional patients to validate the final pop PK model. All eGFRs underrate the true renal clearance in the AKI, so we created pop PK models for VCM dosing in AKI with all eGFRs, to discover that the most accurate model was the one with the Cockcroft-Gault estimator. Since the eGFRs underestimate the true renal clearance in AKI, they are inaccurate for clinical drug dosing decisions, with the exception of the Cockcroft-Gault one, which is appropriate for the pop PK models intended for drug development purposes in AKI.

## 1. Introduction

The glomerular filtration rate (GFR) is computed with estimators (eGFR) that are developed either for chronic kidney disease (CKD) or acute kidney injury (AKI). We adjust the drug dose based on the eGFR in patients with CKD [1]. The GFR estimation is a less accurate but easier method than the measuring of glomerular filtration rate (mGFR) to calculate the GFR in AKI. The estimated GFR, thus, is often used instead of the measured one in clinical care. The CKD-EPI 2009 [2] estimator has replaced others for CKD diagnostics [3], while the Cockcroft and Gault (CG) [4] and the Modification of Diet in Renal Disease (MDRD) study [1,5] estimators still guide drug dosing decisions in CKD [6,7,8]. However, despite clinical and scientific attempts [6,7,8,9,10], the use of these estimators in AKI to guide drug dosing lacks justification.

AKI that results in acute loss of renal function is defined by several alternative classifications: the Risk, Injury, Failure, Loss, End-stage renal disease (RIFLE) criteria [11], the Acute Kidney Injury Network (AKIN) criteria [12], and The Kidney Disease Improving Global Outcomes (KDIGO) criteria [11,12,13]. These classifications define AKI in terms of an increase in serum creatinine, a decrease of GFR or a change in urine output and oliguria duration. The GFR estimation is not a criterion in the last AKI KDIGO classification since accurate estimators have not been developed yet.

The GFR can be calculated by measuring the creatinine clearance (CrCl) with the diuresis of 24 h or the filtration biomarkers, such as inulin, iothalamate, iohexol, diethylenetriaminepentaacetic acid (DTPA) or (chromium-ethylenediaminetetraacetic acid (EDTA) [14,15,16,17] in the AKI [7].

GFR estimators, the MDRD, CG, and the CKD-EPI 2009 were developed in patients with CKD and have shown to be inferior to the gold standard GFR evaluation method—51Cr-EDTA clearance in the AKI [18,19]. These estimators ignore the renal reserve (an ability of a normal kidney to increase the GFR as a response to noxious factors) and assume the equilibrium state of creatinine kinetics. Even though such assumptions are not held in AKI, the eGFR are still used to guide drug dosing in AKI [1,20,21,22,23,24,25].

The eGFR is also used in pharmacokinetic (PK) modelling in renal impairment patients, though no optimal GFR estimator has been recommended for such modelling [26,27,28,29,30].

The first GFR estimator in AKI, constructed by Jelliffe R.W. in 1972 [23], was more accurate compared to the CG and MDRD ones [24]. Later, new GFR estimators, used for the early prediction of delayed renal graft function in AKI [25], were proposed by Chen et al., 2013, and Yashiro et al., 2012. As far as we know, no comparative analysis has been performed using the CKD and AKI GFR estimators to predict the PK profiling of a renally excreted drug vancomycin (VCM, 80% of VCM is excreted unchanged in the urine) in AKI [27,30], so we aimed to compare the performance of different GFR estimators in building PK models to predict a mainly renally excreted drug concentration in patients with AKI (Appendix A).

## 2. Materials and Methods

### 2.1. Study Population

We included patients in the study who were hospitalized in any department of LUHS Kaunas Clinics, were at least 18 years old, had received VCM and had developed a non-VCM-induced AKI and had experienced an increase in serum creatinine (SCr) by ≥0.3 mg/dl (≥26.5 mcmol/L) within 48 h or an increase in SCr to ≥1.5 times from baseline within the prior 7 days or had experienced oliguria (defined as urine volume < 0.5 mL/kg/h for 6 h) [13]. No patients were included in this study with VCM-induced kidney injury. Patients who were on haemodialysis or had chronic renal failure were excluded from this study.

### 2.2. Ethical Statement

We performed the study by following the Declaration of Helsinki and its amendments [28], and all study protocols were approved and permitted by the independent Kaunas Regional Biomedical Research Ethics Committee (P3-BE-2-35/2013).

### 2.3. Drug Analysis

We measured the VCM serum concentrations with the turbidimetric inhibition immunoassay method [29] before the second administration of VCM and did not reduce the first dose of 15 mg/kg VCM by the degree of renal injury. We included only the VCM trough concentrations that were obtained no more than 1 h before the administration of VCM in the analysis.

### 2.4. The Individual PK Models Based on the Single-Dose Intermittent Infusion (SDII) Model

We described the SDII model with the following equation [30]:

The single-dose intermittent infusion (SDII) model:

C = [k_0_/(k_e_ × V)]×(1 − e^−k^_e_^t′^) × (e^−k^_e_^t^) (1)

C (mg/L)—predicted VCM serum concentration at time = t,

V—volume of distribution;

k_e_—elimination rate constant—a relationship between renal and VCM elimination: k_e_ = Cl/V, Cl = 0.695(CrCl) + 0.05 [30];

CrCl—creatinine clearance in mL/min as estimated using the MDRD, CKD-EPI 2009, Chen et al. [26] and Yashiro et al. [10] equations, adjusted to body surface area;

k_0_—the infusion rate (expressed in amount per unit time as mg/h);

t′—infusion time;

t—time.

We calculated the mean prediction error (MPE%) and mean absolute prediction error (MAE%) to assess the individual PK models.

### 2.5. Statistical Analysis

We summarized data with descriptive statistics as mean (standard deviation), identified monotonic associations among variables with Spearman’s correlation coefficient or identified the strength of the linear relationship among variables with Pearson’s correlation if the data were symmetrically distributed. We used analysis of variance to compare the means across the groups with a post hoc Tukey-Kramer adjustment if the assumptions regarding the data distribution were not violated [31]; we deemed the results to be statistically significant when *p* < 0.05.

### 2.6. Populiation PK Model Development

Using the data of 34 patients, we developed a base model with fixed effects with no covariates to identify the population volume of distribution and clearance values to predict the VCM concentration. We constructed separate one-compartment infusion models (with one random effect) with each of the GFR estimates and used the latter as covariates for clearance [30,32]. After establishing the optimal covariate model, we used weight as a covariate for the volume of distribution in the models and included two random effects for the population volume of distribution and clearance. We used the first-order integration method of the Beal and Sheiner and dual quasi-Newton optimization technique to fit the one-compartment pop PK model as implemented in SAS^®^ University Edition PROC NLMIXED and conducted the NLMIXED procedure by carefully following the SAS documentation, as provided by the SAS Institute Inc. The model with the lowest objective function value (negative log-likelihood), model fit criteria −2 log-likelihood and Akaike information criterion (AIC) was deemed to be the most appropriate. We used the correlation between the observed and predicted values to diagnose the pop PK models. We assessed the accuracy and precision by computing the mean prediction error (MPE%, Equation (2)) and mean absolute prediction error (MAE%, Equation (3)) of the final model to evaluate the goodness of fit of the model. In equations 2 and 3, the ${\mathrm{Obs}}_{i}$ is the observed drug concentration given some dose at time t and ${\mathrm{Pred}}_{i}$ was the predicted concentration at the same dose and time [33]. Six additional patients were included in the study to test the final pop PK model.

We built one-compartment VCM models since they are non-inferior to two- and three-compartment models [29,34]:

Mean prediction error:

(2) $$\mathrm{MPE}\%=\frac{1}{N\;}\sum_{i=1}^{N}(\frac{{\mathrm{Obs}}_{i}-{\mathrm{Pred}}_{i\;}}{{\mathrm{Obs}}_{i}})’$$

where $\frac{{\mathrm{Obs}}_{i}-{\mathrm{Pred}}_{i\;}}{{\mathrm{Obs}}_{i}}$ is prediction error (PE).

Mean absolute prediction error:

(3) $$\mathrm{MAE}\%=\frac{1}{N\;}\overset{N}{\underset{i=1}{\sum }}|(\frac{{\mathrm{Obs}}_{i}-{\mathrm{Pred}}_{i\;}}{{\mathrm{Obs}}_{i}})|$$

We defined the outlier as an observation with the outlier z score > 0.5 [35] and removed them before constructing the population PK models.

Outlier definition:

Outlier z score = (OV_i_ − mean(OV))/SD(OV), where OV = D/C/dT. (4)

OV—outlier value, 

OV_i_—OV value of i-th observation,

D—dose administered, 

C—concentration, 

dT—delta time between administration and concentration measurement.

We estimated the eGFR of patients with the CG, MDRD, CKDEPI, kinetic GFR estimation by Chen et al. (Ckegfr) ([28]) and Yashiro et al. (Ykegfr) [10] and Jelliffe and Jelliffe (JJGFR) estimators [23].

## 3. Results

**Figure 1:**
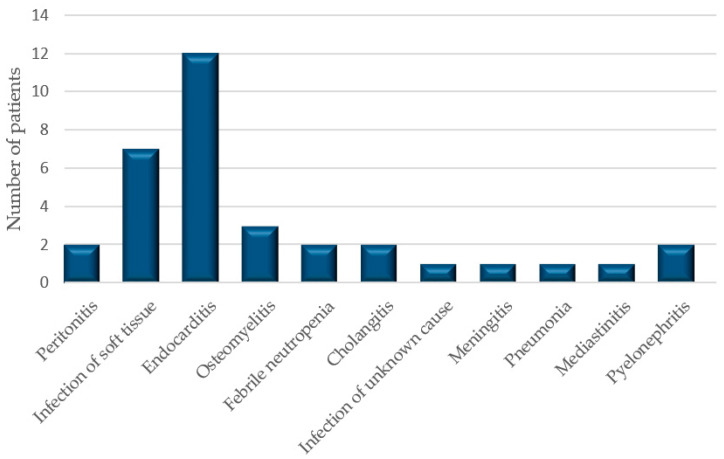
The diagnoses of patients.

After screening 163 patients treated with vancomycin between 1 January 2016 and 1 January 2017, who had their serum creatinine measured, we identified 40 of them (26 men and 14 women) with AKI and simultaneous VCM administration. The pop PK model was developed by using the 34 patient (24 men and 8 women) sparse sampling data. The remaining six subjects were used to test the final pop PK model. The baseline demographics of the patients are in Figure 1 and Table 1. 

**Figure 2:**
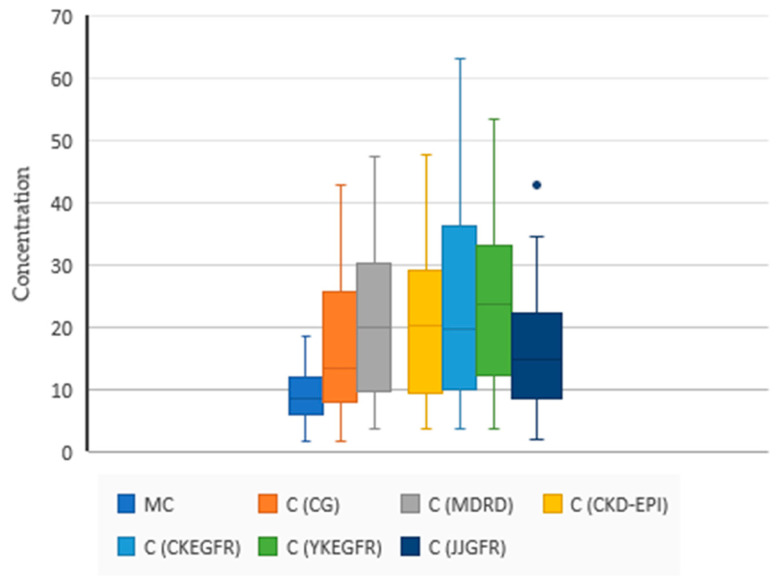
Summary statistics of the predicted (C) and measured concentrations (MC) of VCM with individual PK models based on different GFR estimators. N = 34, MC 9.31 (4.17), C (CG) 17.17 (10.74), C (MDRD) 22.12 (13.35), C (CKD-EPI) 22.08 (13.19), C (CKEGFR) 24.07 (15.30), C (YKEGFR) 24.39 (13.27), C (JJGFR) 16.23(9.66). The blue dot is an outlier. One-way ANOVA (F (4165) = 2.1398, *p* = 0.078).

The summary statistics and Spearman’s correlation between the predicted (using SDII models) and measured vancomycin concentrations are presented in Table 2, Figure 2 and Table 3. 

The SDII model based on the CG had an MPE% of 157.70 and MAE% of 184.09, and the SDII model based on JJGFR had an MPE% of −137.76 and MAE% of 164.31. Other models were even less accurate: the MDRD-based model had an MPE% of −258.48 and MAE% of 279.25, the CKD-EPI based model had an MPE% of −276.02 and MAE% of 295.30, the KEGFR-based model had an MPE% of −276.02 and MAE% of 295.30 and the YKEGFR-based model had an MPE% of −278.32 and MAE% of 292.42. The lowest MPE% and MAE% values were found for the CG and JJGFR methods. The mean time between creatinine measurements was 40.35 (17.17) hours. The first creatinine measurement was taken before the VCM administration, and the second creatinine measurement was taken before the second VCM administration. Based on MPE% and MAE% findings for different GFR estimations, we considered that the best model should be constructed with the CG estimate used as a covariate of clearance and the body weight as a covariate for the volume of distribution. We constructed the following nonlinear mixed-effect model to predict the VCM plasma concentrations in patients with AKI. The final model is the following one:

population model one-compartment model [36]:

Predicted concentration = dose/vol × exp(−1 × (CL/Vol) × time),(5)

where CL = exp (−5.5079 + 0.01593 × CG (mL/min)), vol = exp (2.8501 + 0.01306 × weight (kg)). 

Concentration-predicted VCM plasma concentration, with the following abbreviations:

Cl—clearance, 

dose—administered VCM dose, 

time—the time between administration and concentration measurement, 

Vol—volume of distribution.

Population model predictions were compared with the measured VCM concentrations (Table 4 and Table 5) to assess the model suitability.

### Population PK Model Assessment

We used the mean prediction error (MPE%) and the mean absolute prediction error (MAE%) to assess the model goodness of fit in six patients; the MPE% was −16.91 and the MAE% was 24.47 [33].

We found an article where a VCM pop PK model was built and two clinical cases were used to validate it and compared our pop PK model to the published one [33]. 

Case 1. A 69-year-old woman, 65 kg, was prescribed VCM every 12 h as an IV infusion. The CG rate was 40.6 mL/min (serum creatinine value 118.1 μmol/L). The model’s predictions were 8.5, 12.8, 17.0, 21.3 and 25.5 mg/L when prescribing an IV infusion of 250, 500, 750, 1000, 1250 and 1500 mg VCM every 12 h, whereas our estimates were 5.32, 10.63, 15.95, 21.27 and 26.59 mg/L, respectively. The 15–20 mg/L was determined to be the target therapeutic concentration. In this specific case, a 1000 mg VCM dose every 12 h was chosen. In the result, two days after the beginning of dosing, the trough concentration of VCM was 15.7 mg/L with 8.3% prediction error (PE); our model prediction was 35.5% PE.

Case 2. A 62-year-old man, 68 kg, was prescribed VCM every 12 h, CG 45.7 mL/min, the predictions were 7.8, 11.7, 15.7, 19.6 and 23.5 mg/L, when prescribing IV infusions of 500, 750, 1000, 1250 and 1500 mg VCM every 12 h. Our model predicted 10.14, 15.02, 19.78, 24.42 and 28.94 mg/L. The patient’s blood samples were obtained 2 days after the VCM administration, and the concentration was 26.0 mg/L, with 39.6% PE; our prediction had −23.9% PE.

## 4. Discussion

We showed that individual PK models with the eGFR overestimate the VCM concentrations in patients with AKI and, thus, underestimate the true renal clearance. The latter occurs because the tubular secretion of creatinine and other elimination pathways in the AKI, that differ from the ones seen in the CKD [37], are ignored. It was previously shown that calculating GFR CG, MDRD and the CKD-EPI 2009 estimators in the AKI may be inaccurate [17,38]. We expected that the equations specifically designed for AKI (JJGFR [23], YKEGFR [10] and CKEGFR [26]) could outperform other GFR estimators designed for CKD. However, the individual PK models based on AKI-specific GFR estimators did not outperform the models based on the CKD-specific GFR estimators. We drew this conclusion by keeping in mind that the individual PK models have shortcomings: they do not include covariates to explain the population variation of the PK parameters and require intensive sampling to get optimal results [39,40,41,42,43,44]. In this research we assessed the GFR by approximating it with VCM clearance in patients with AKI. This strategy has a limitation because nonrenal clearance of VCM in the AKI makes it a less reliable marker for renal function than GFR assessment with inulin [45]. We also compared the performance of the GFR estimators in endocarditis patients with stable renal function and gentamicin (Appendix B) and found that all estimators were fairly accurate, while the observed differences in accuracy were marginal. The use of “estimators” of GFR can only work in a relatively stable situation. In other words, these estimations in intensive care units (ICUs) will hardly ever work because of the dynamic fluctuation of renal function.

The CG and JJGFR estimators seem to be more appropriate for building the pop PK models when compared to other estimators. We deemed the CG to be the preferred method for GFR estimation in pop PK modelling due to its simplicity. However, as far as drug dosing is concerned, the best approach still seems to be 24-h urine collection or the use of filtration markers (i.e., EDTA, iothalamate and iohexol) to calculate the creatinine clearance [16,17].

Our final population model included weight and creatinine clearance based on CG as covariates. Inclusion of these covariates is a common practice because other parameters such as age and sex were indirectly accounted for by the CG [27]. The pop PK models with the AKI-specific GFR estimators did not outperform the CKD-specific GFR estimators in the AKI patients. By developing these models, we show that different GFR estimators yield different results and that AKI-specific GFR estimators do not increase model performance. Despite these results, a tribute ought to be paid to authors that developed the idea of non-stable GFR, especially Chen et al., who defined the problem of estimation of unstable kidney function in clinical practice in a very clear and eloquent fashion without “the necessity to dwell into arcane mathematical notation” as said by himself [26]. Our study shows that AKI-specific eGFR methods do not aid in the development of pop PK models, complicate the development of such models and are not accurate enough to guide drug dosing in AKI patients.

## 5. Limitations

The use of “estimators” of GFR can only work in a relatively stable situation. In other words, these estimations in intensive care units (ICUs) will hardly ever work because of the dynamic fluctuation of renal function.

## 6. Conclusions

GFR estimation is inaccurate in the patients with AKI. GFR estimators are appropriate to use in pop PK.

## Figures and Tables

**Table 1:** The baseline demographics of patients (the Cockcroft–Gault (CG) estimator, the modification of diet in renal disease (MDRD) estimator, the chronic kidney disease epidemiology collaboration (CKD-EPI) equation, the kinetic glomerular filtration rate (GFR) estimator by Chen (CKEGFR), estimation of creatinine clearance before steady state according to Yashiro (YKEGFR), the Jelliffe and Jelliffe estimator (JJGFR)).

Variable	Mean (SD)
Age in years	62.19 (15.61)
Height in cm	172.76 (9.10)
Weight in kg	87.65 (25.91)
Creatinine level in µmol/L	263.91 (204.73)
CG	41.77 (27.19)
MDRD	30.35 (22.27)
CKDEPI	30.48 (21.84)
CKEGFR	29.34 (19.92)
YKEGFR	25.42 (16.63)
JJGFR	44.36 (30.71)

**Table 2:** Summary statistics of the predicted (C) and measured concentrations (MC) of vancomycin (VCM) with individual pharmacokinetic (PK) models based on different GFR estimators.

Variable	N	Mean	Std Dev	Median
MC	34	9.31	4.17	8.65
C (CG)	34	17.17	10.74	13.47
C (MDRD)	34	22.12	13.35	20.02
C (CKD-EPI)	34	22.08	13.19	20.24
C (CKEGFR)	34	24.07	15.30	19.67
C (YKEGFR)	34	24.39	13.27	23.68
C (JJGFR)	34	16.23	9.66	14.77

**Table 3:** Correlation of the predicted (C) and measured concentrations (MC) with different methods to estimate kidney function with individual PK models with the single-dose intermittent infusion (SDII) equation (Spearman correlation coefficients with representative *p* values, N = 34), sig. = *p* < 0.0001. Red color indicates positive correlation; blue color indicates negative correlation.

	MC	C (CG)	C (MDRD)	C (CKD-EPI)	C(KEGFR)	C(YKEGFR)	C (JJGFR)
MC	1.00	−0.20	−0.38	−0.35	−0.29	−0.34	−0.20
		0.25	0.03	0.04	0.09	0.05	0.26
C (CG)		1.00	0.92	0.94	0.88	0.91	0.96
			sig.	sig.	sig.	sig.	sig.
C (MDRD)			1.00	0.99	0.91	0.96	0.88
				sig.	sig.	sig.	sig.
C (CKDEPI)				1.00	0.91	0.96	0.90
					sig.	sig.	sig.
C(KEGFR)					1.00	0.85	0.83
						sig.	sig.
C(YKEGFR)						1.00	0.93
							sig.
C (JJGFR)							1.00
							0.94

**Table 4:** Developed population pharmacokinetic models, *n* = 34.

Population Model	Negative Log Likelihood	−2 Log Likelihood	AIC	Pop. Clearance (L/min)	SE	*p*	Pop. Volume of Distribution (L)	SE	*p*
Base model	128.94	257.9	267.9	0.007	0.002	0.001	32.81	32.81	0.706
Model with CG	125.55	251.1	263.1	0.004	0.002	0.021	17.29	7.24	0.024
Model with MDRD	126.58	253.2	265.2	0.007	0.003	0.007	22.62	9.26	0.021
Model with CKDEPI	126.60	253.2	265.2	0.007	0.003	0.009	22.66	9.36	0.022
Model with CKEGFR	126.57	253.1	265.1	0.007	0.002	0.005	22.55	9.27	0.022
Model with YKEGFR	126.48	253.0	265.0	0.008	0.003	0.009	22.16	8.86	0.018
Model with JJGFR	126.38	252.8	264.8	0.005	0.003	0.075	19.25	9.55	0.054
Model with 2 random effects based on CG	140.98	282.0	298.0	0.091	<0.001	0.001	7.91	0.002	<0.001

**Table 5:** Pearson’s correlation between observed vs. predicted vancomycin concentrations with different methods to estimate the GFR in pop PK models, *n* = 34.

Pop PK Models	MC Mean (SD)	PC Mean (SD)	Pearson’s Correlation r	*p*
Base model	13.64 (6.38)	13.13 (3.94)	−0.15	0.336
Model with CG	13.64 (6.38)	13.14 (4.65)	0.63	<0.001
Model with MDRD	13.64 (6.38)	13.20 (4.13)	0.15	0.342
Model with CKDEPI	13.64 (6.38)	13.23 (4.09)	0.15	0.345
Model with CKEGFR	13.64 (6.38)	13.21 (4.13)	0.16	0.339
Model with YKEGFR	13.64 (6.38)	13.16 (4.25)	0.15	0.342
Model with JJGFR	13.64 (6.38)	13.14 (4.65)	0.55	<0.001
Model with 2 random effects and CG	13.64 (6.38)	14.07 (11.69)	−0.17	0.29

## Data Availability

The data, material and code will be provided upon request.

## Appendix A

Creatinine clearance estimators in AKI

Kinetic GFR estimator by Chen (2013) [26]:

Creatinine clearance equations for estimation of GFR in AKI:

(A1) $$\mathrm{KeGFR}=\frac{\mathrm{SSPcr}\times \mathrm{CrCl}}{\mathrm{MeanPcr}}\times \left(1-\frac{24\times \Delta \mathrm{Pcr}}{\Delta \mathrm{Time}\left(h\right)\times \mathrm{Max}\Delta \mathrm{Pcr}/\mathrm{Day}}\right)$$

SSPcr—steady-state plasma creatinine; in our research, it was assumed that there is small discordance between serum and plasma creatinine level estimation [41].

CrCl—creatinine clearance estimated with the CG formula [4].

MeanPCr is the equivalent of PCr in the clearance equation because the kinetic situation deals with two creatinine points: the starting and the ending values. The arithmetic mean yields a single halfway value that is suitable for use in the clearance equation.

∆Pcr—change in plasma creatinine.

∆Time (h)—interval in hours between two consecutive creatinine measurements.

Max∆Pcr/Day—the maximal change (increase) in the plasma creatinine that can occur per day if renal function is completely lost (1.0–1.5 mg/dl per day, we used 1.5 mg/dL as standard [26]).

Estimation of creatinine clearance before steady state according to Yashiro (2012) [10]:

(A2) $$\mathrm{CrCl}=\frac{\left\{G-V\times \frac{\left({\mathrm{Cr}}_{2}-{\mathrm{Cr}}_{1}\right)}{\left(t_{2}-t_{1}\right)}\right\}}{\left\{\frac{\left({\mathrm{Cr}}_{1}+{\mathrm{Cr}}_{2}\right)}{2}\right\}}$$

CrCl—estimated creatinine clearance in mL/min/1.73 m^2^.

G—creatinine generation rate (G) (mg/min) defined as:

$$G=1.258\times {\mathrm{Cr}}^{-0.094}\times {\mathrm{age}}^{-0.287}\left(\times 0.739\;\mathrm{if}\;\mathrm{female}\right)$$

V—a volume of body fluid (assumed as 60% of body weight (BW)).

Cr_1_—first measured creatinine serum concentration, at time t_1_.

Cr_2_—second measured creatinine serum concentration, at time t_2_.

The Jelliffe and Jelliffe estimator [9,23,42]:

CrCl (in mL/min/1.73 m^2^) = E/(14.4⋅Scr_ave_) (A3)

CrCl—estimated creatinine clearance in mL/min/1.73 m^2^.

E—Ess_corrected_.

Scr_ave_—average of the two serum creatinine determinations in mg/dL.

Ess_male_ = IBW [29.3 − (0.203⋅age)]

Ess_female_ = IBW [25.1 − (0.175⋅age)]

Ess—the excretion of creatinine.

IBW—ideal body weight in kilograms.

Age—age in years.

Ess_corrected_ = Ess [1.035 − (0.0337⋅Scrave)]

Scr_ave_—an average of the two serum creatinine determinations in mg/dL.

(A4) $${\mathrm{Ess}}_{\mathrm{corrected}}-\frac{\left[4\times \mathrm{IBW}\left({\mathrm{Scr}}_{1}-{\mathrm{Scr}}_{2}\right)\right]}{\Delta t}$$

Scr—the first serum creatinine in mg/dL.

Scr_2_—the second serum creatinine in mg/dL.

Δt—the time between the measurement of Scr_1_ and Scr_2_ in minutes.

The steady-state creatinine clearance estimators.

The Cockcroft–Gault (CG) estimator [4]:

Ccl = [140 − age (years)] × [body weight(kg)]/72 × Serum Cr (mg/dL) (A5)

Ccl—creatinine clearance, (15% less in women).

The modification of diet in renal disease (MDRD) estimator [43] for

eGFR (mL/min/1.73 m^2^) = 175 × Cr − 1.154 × Age − 0.203 × 0.742 (if female) (A6)

The chronic kidney disease epidemiology collaboration (CKD-EPI) equation:

eGFR (mL/min/1.73 m^2^) = 141 × min (Scr/K, 1)^α^ × max (Scr/K, 1)^−1.209^ × 0.993 Age × 1.018 (if female). (A7)

## Appendix B

### Appendix B.1. Supplementary Study in Patients with Endocarditis

We assessed the performance of eGFR estimators in patients with endocarditis receiving gentamicin with chronic kidney disease or normal renal function. Gentamicin is mainly renally excreted via glomerular filtration—65% to 100% [1]—and so kidney dysfunction is the most important factor influencing gentamicin pharmacokinetics [2,3]; thus, the performance of GFR estimators can be assessed by comparing gentamicin concentration PK models based on different GFR estimators.

### Appendix B.2. Methods

Anonymized medical records of 61 patients receiving gentamicin for the treatment endocarditis at the Limoges University Hospital (France) were screened and data collected during a 1-year period, and single-dose intravenous infusion (SDII) models were constructed to predict gentamicin concentrations.

Gentamicin concentrations were determined in the Pharmacology Department of Limoges University Hospital using a validated immunoassay method (Architect^®^ ci4100, Abbott core).

The work described was carried out in accordance with the Code of Ethics of the World Medical Association (Declaration of Helsinki).

Descriptive statistics were presented as mean, standard deviation and 95% confidence interval for mean. If a variable was not distributed normally and was asymmetric, it was represented with the median (Md) and 1st and 3rd quartile (Q1; Q3). Linear regression analysis was used to identify the SDII model that predicted the measured gentamicin concentrations most accurately.

The relationship between renal and aminoglycoside elimination (Hull JH, Sarubbi FA, 1976; Sarubbi FA, Jr., 1978) was defined as follows:

ke (in h^−1^) = 0.00293 × (CrCl in mL/min) + 0.014

CrCl is creatinine clearance, adjusted for body surface area.

Ke—gentamicin elimination rate constant.

### Appendix B.3. Results

The patient age mean was 70.51 (with standard deviation 13.33), body surface area was 1.91 (0.29).

The estimated GFR based on MDRD was Md = 74.04 (55.87; 113.54), based on CKD-EPI 74.76 (55.19; 100.24), CKEGFR 79.38 (63.31; 108.22), YKEGFR 57.96 (44.86; 86.19) JJGFR 70.62 (48.84; 102.97).

A simple linear regression was calculated to predict measured gentamicin concentration based on predicted gentamicin concentration as estimated using SDII models with different eGFR equations.

SDII model based on Chen eGFR equation:

b = 1.454, 95% Confidence Interval (1.208, 1.700), t (59) = 11.821, *p* < 0.001, R2 = 0.703, F (1, 59) = 139.733, *p* < 0.001.

SDII model based on Yashiro eGFR equation:

b = 1.375, 95% Confidence Interval (1.180, 1.571), t (59) = 14.078, *p* < 0.001, R2 = 0.771, F (1, 59) = 198.198, *p* < 0.001.

SDII model based on MDRD equation:

b = 1.429, 95% Confidence Interval (1.196, 1.662), t (59) = 12.285, *p* < 0.001, R2 = 0.719, F (1, 59) = 150.917, *p* < 0.001

SDII model based on CKDEPI equation:

b = 1.417, 95% Confidence Interval (1.192, 1.643), t (59) =12.585, *p* < 0.001, R2 = 0.729, F (1, 59) = 158.389, *p* < 0.001

SDII model based on Jelliffe and Jelliffe equation:

b = 1.265, 95% Confidence Interval (1.003, 1.527), t (59) = 9.660, *p* < 0.001, R2 = 0.613, F (1, 59) = 93.317, *p* < 0.001

### Appendix B.4. Bibliography

1. Fresenius Kabi USA L (per D (2013) Product Information: gentamicin intramuscular injection solution, intravenous injection solution, gentamicin intramuscular injection solution, intravenous injection solution. Available online: https://www.accessdata.fda.gov/drugsatfda_docs/label/2014/062366s033lbl.pdf (accessed on 25 July 2019).
2. Barza, M.; Brown, R.B.; Shen, D.; Gibaldi, M.; Weinstein, L. Predictability of blood levels of gentamicin in man. *J. Infect. Dis.* **1975**, *132*, 165–174.
3. Kaye, D.; Levison, M.E.; Labovitz, E.D. The unpredictability of serum concentrations of gentamicin: pharmacokinetics of gentamicin in patients with normal and abnormal renal function. *J. Infect. Dis.* **1974**, *130*, 150–154.
